# Upstream sequence elements direct post-transcriptional regulation of gene expression under stress conditions in yeast

**DOI:** 10.1186/1471-2164-10-7

**Published:** 2009-01-07

**Authors:** Craig Lawless, Richard D Pearson, Julian N Selley, Julia B Smirnova, Christopher M Grant, Mark P Ashe, Graham D Pavitt, Simon J Hubbard

**Affiliations:** 1 Michael Smith Building, Faculty of Life Sciences, University of Manchester, Manchester, M13 9PT, UK; 2 School of Computer Science, University of Manchester, Oxford Road, Manchester, M13 9PL, UK; 3 Wellcome Trust Centre for Human Genetics, University of Oxford, Roosevelt Drive, Oxford OX3 7BN, UK

## Abstract

**Background:**

The control of gene expression in eukaryotic cells occurs both transcriptionally and post-transcriptionally. Although many genes are now known to be regulated at the translational level, in general, the mechanisms are poorly understood. We have previously presented polysomal gradient and array-based evidence that translational control is widespread in a significant number of genes when yeast cells are exposed to a range of stresses. Here we have re-examined these gene sets, considering the role of UTR sequences in the translational responses of these genes using recent large-scale datasets which define 5' and 3' transcriptional ends for many yeast genes. In particular, we highlight the potential role of 5' UTRs and upstream open reading frames (uORFs).

**Results:**

We show a highly significant enrichment in specific GO functional classes for genes that are translationally up- and down-regulated under given stresses (e.g. carbohydrate metabolism is up-regulated under amino acid starvation). Cross-referencing these data with the stress response data we show that translationally upregulated genes have longer 5' UTRs, consistent with their role in translational regulation. In the first genome-wide study of uORFs in a set of mapped 5' UTRs, we show that uORFs are rare, being statistically under-represented in UTR sequences. However, they have distinct compositional biases consistent with their putative role in translational control and are more common in genes which are apparently translationally up-regulated.

**Conclusion:**

These results demonstrate a central regulatory role for UTR sequences, and 5' UTRs in particular, highlighting the significant role of uORFs in post-transcriptional control in yeast. Yeast uORFs are more highly conserved than has been suggested, lending further weight to their significance as functional elements involved in gene regulation. It also suggests a more complex and novel mechanism of control, whereby uORFs permit genes to escape from a more general attenuation of translation under conditions of stress. However, since uORFs are relatively rare (only ~13% of yeast genes have them) there remain many unanswered questions as to how UTR elements can direct translational control of many hundreds of genes under stress.

## Background

Much of the focus of post-genome science is now switching from simply cataloguing genes and gene products, to the greater challenge of understanding how they interact and regulate one another. The regulation of gene expression underpins the ability of cells to adapt to environments, deal with stresses, and progress through reproductive and cell cycle changes. Similarly, the increased complexity by which higher eukaryotes can control and manage the levels of their gene products has been suggested to underlie complexity in higher organisms – a compelling argument when the small difference between the numbers of genes in humans and the fly is considered. Yet a detailed understanding of how even the simplest cell controls its gene complement remains elusive, though great strides are being made in the field of systems biology. Much of this research has exploited advances in array-based technologies to characterise how gene expression levels change during the cell cycle or in response to stresses or environmental changes [1,2], allowing transcriptional factors to be mapped to the genes they regulate [3,4]. However, these approaches only deliver regulatory control information at the transcriptional level. The production, maturation and export of mRNA from the nucleus precedes translation into the gene products (proteins) but this latter process can also be regulated by a variety of post-transcriptional control mechanisms [5-14] and the measured correlations between transcriptome and proteome levels are imperfect [e.g. [15]] Most studies observe strong, but imperfect, correlations ranging from 0.2–0.8, suggesting that additional control at the mRNA or protein level must be present. Indeed, it has been suggested that as little as 20–40% of the control on gene expression can be attributed to mRNA levels [12,16]. Many of these post-transcriptional control processes are governed by the sequence and structure of the mature mRNA molecule, affecting the regulatory molecules (usually proteins) that can interact with the transcript. Eukaryotic mRNA molecules have a tripartite structure, with a 5' untranslated region (UTR) preceding the principal open reading frame (ORF) and followed by a 3' UTR. UTRs are critical to the post-transcriptional regulation of gene expression, modulating mRNA export from the nucleus and affecting translational efficiency, subcellular localization and mRNA stability [17]. Furthermore, mutations in UTRs can lead to serious pathology [18], demonstrating their importance to proper function in the cell.

Previous work from our laboratories has examined the effects that different stresses have on both the transcriptional and translational regulation of genes in the model organism, yeast [19,20]. Gene arrays were used to monitor changes in transcript abundance for RNA populations from both stressed and control cells, which were additionally combined with polysomal gradient analyses to investigate the effects of different stress conditions on the yeast genome. The first study demonstrated a general down-regulation of translation and protein synthesis, where different RNA subsets depending on the given stress could resist this general trend and were regulated translationally [19]. A second yeast study considered oxidative stresses which also elicit complex and different translational reprogramming effects, even at different concentration of hydrogen peroxide [20]. These data provide excellent test sets of genes to examine potential translational regulation signals which could single out these genes for specific targeting at the translational level. Until recently however, the true transcriptional start sites (TSSs) of many yeast genes were unknown, making it difficult to quantify the effects of UTRs on post-transcriptional regulation. However, several recent large-scales studies using 5' SAGE [21] and G-Capping cDNAs [22] have defined TSSs for 2231 and 3599 yeast genes respectively. In addition, tiling array studies have characterised complete transcripts at both the 5' and 3' end for over a third of the yeast genome [23].

Here we examine the properties of these characterised yeast UTRs in the context of the stress-response microarray data sets [19,20], looking for UTR properties (length, uORF content and conservation) which determine differential regulation. In particular we examine upstream open reading frames (uORFs), which can affect the level of gene expression *via *a number of potential post-transcriptional mechanisms [5,6,24-26] These include mRNA degradation *via *the nonsense mediated decay pathway (NMD) [26], possible activities of the small peptides encoded by the uORF themselves [25], and mechanisms relating to the scanning of the transcript by the ribosome. One such mechanism is "leaky scanning" where a proportion of the scanning complexes bypass the uORF AUG and continue scanning the transcript onto a downstream start codon. In this case, the uORF AUG acts as a "decoy" from the standard AUG start, so that it acts as a negative regulator of gene expression at least for some fraction of ribosomes. Similarly, the context of an ORF or uORF stop codon can exert general effects on translation by modulating the ability of the ribosome to reinitiate after termination and translate downstream ORFs [26]. This latter case has been studied in great detail for the GCN4 UTR where several uORFs combine to produce complex regulatory effects under stress, chiefly involving differential behaviour towards uORFs 1 and 4 [14]. Similarly, the uORFs in *YAP1 *and *YAP2 *have been studied in detail, revealing different potential mechanisms for destabilising mRNA post-termination after ribosomal scanning [6]. As already mentioned, uORFs have also been implicated in the nonsense mediated decay (NMD) pathway, where aberrant transcripts are removed and it has been suggested that uORFs may be translated and trigger NMD in as many as 35% of cases [26]. Moreover, several studies have looked at conservation and functional significance in yeast [6,27,28], and more recently flies [29], concluding that a limited number of uORFs other than *GCN4 *appear to be conserved and are functionally operative.

Our results provide further evidence that not only does yeast exert a general, large scale control over its genes at the translational level, but that under stress this effect is controlled by UTR sequence elements. This is the first evidence that suggests UTR elements are specifically responsible for translational control of an experimentally-determined gene set which is differentially expressed under stress. Although rare, we propose that uORFs play a role in this regulation for a significant number of genes, not just restricted to a very limited subset (e.g. *GCN4, YAP1/2*). We show that uORF stop codon readthrough is likely and propose that uORF-mediated changes to reinitiation competency is a common mechanism to regulate gene expression under stress, consistent with that proposed for *GCN4*. Nevertheless, although uORFs provide some explanations, translational control is apparently both widespread and complex, and other mechanisms must be responsible for the post-transcriptional control of the majority of yeast genes which exploit it.

## Results and Discussion

### Defining 5'and 3'UTRs

**Figure 1:**
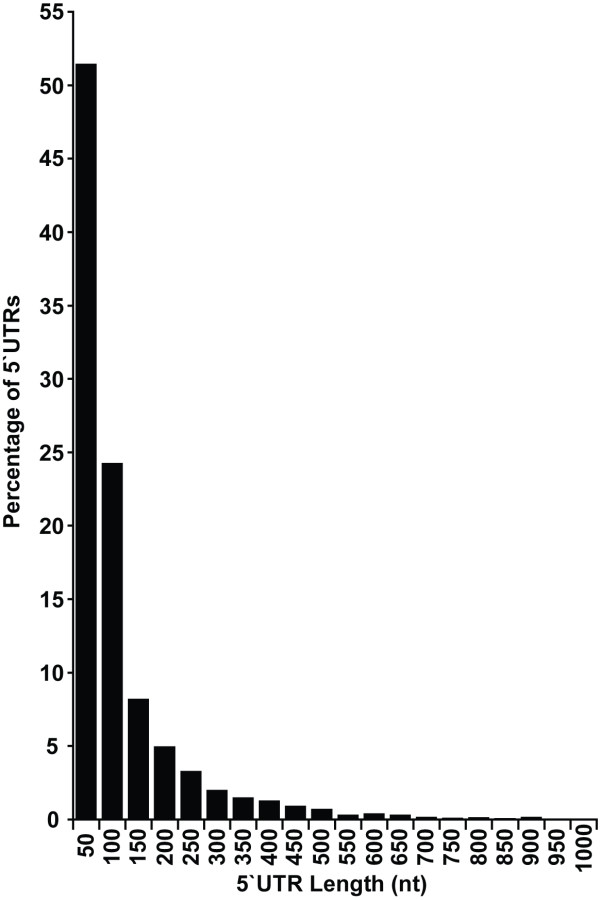
**Length distribution of yeast 5' UTRs**. Data was taken from a superset of transcriptional start sites, selecting one per gene. The majority are less than 50 nucleotides in length.

The two sets of Transcriptional Start Sites (TSS) from the GCapping and 5'SAGE method were combined to create a 5' TSS superset. This resulted in a total of 53,673 individual TSS determinations for 4,149 yeast ORFs which contains considerable redundancy, with many repeated observations. To remove redundancy and maximize coverage, a single TSS was selected for each yeast ORF using a simple selection rule, taking the most abundant TSS position listed, or in the case of ambiguity, the longest UTR. All UTR introns were excised from relevant UTR sequences. The UTR intron co-ordinates were obtained from the *Saccharomyces *Genome Database [30]. Figure 1 shows the length distribution of the TSS superset, which have a mean length of 89 nt. Although there are some longer, characterised UTRs, they are generally short and most are 50 nt or less. A further recent study used tiling arrays to define both 5' and 3' ends for RNAs with polyA tails corresponding to 2,044 genes [23]. The tiling array 5' UTR lengths are broadly in agreement between this and our current dataset (correlation coefficient r = 0.56) although there is no significant correlation between 5' and 3' UTR length (r = 0.10) (see also Additional File 1).

### Identification of uORFs

**Figure 2:**
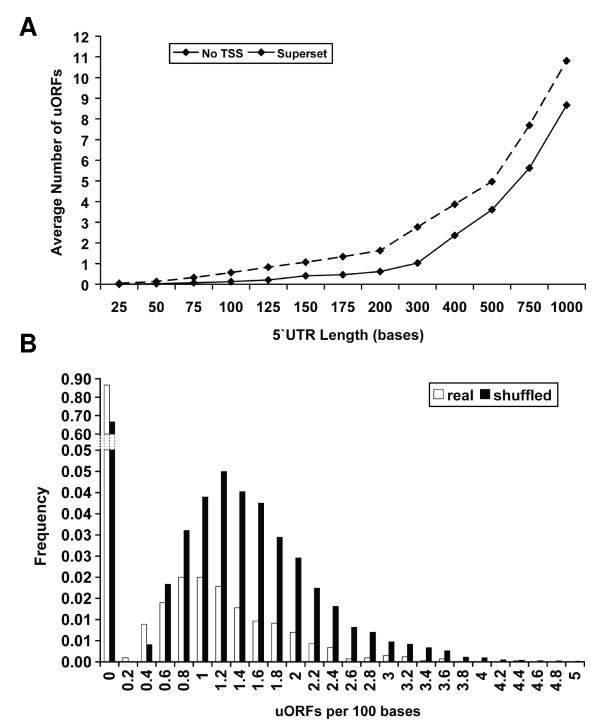
**Statistical significance in yeast upstream sequences and 5' UTRs**. In A), the average number of uORFs is shown for both upstream regions in general (No TSS) and TSS mapped 5' UTRs (Superset). The same number of sequences (4149) is used in all cases for the "No TSS" line. At all points, there are fewer uORFs in the real 5' UTR sequences, In B), a normalised frequency histogram of uORF numbers in real and shuffled 5' UTRs highlights the reduced chance of observing a uORF in real UTR sequence compared to random. Over 85% have none compared to 68% by chance.

The selected 5'UTR sequences for all 4149 ORFs were submitted to a uORF identification procedure. Identified uORFs had to conform to the following rules: start at least 20 nt from the 5' mRNA end to take into account the 5'cap, be minimally 9 nt in length (a start codon, another codon and a stop), and not extend more than 100 nt into the principal ORF. Generally, uORFs are relatively rare; a total of 1481 uORFs were identified in 554 UTRs, corresponding to 13% of the yeast ORFs in the dataset. Only 182 of these uORFs extended into the principal ORF. Figure 2A shows the average number of uORFs contained within upstream segments of increasing distance from the AUG start codon where the true TSS is ignored; these segments will therefore extend upstream beyond the true 5' start into intergenic sequence in many cases. This is compared to the superset of TSS defined 5' UTRs from two recent studies [21,22]. The results in Figure 2A show the average number of uORFs increases when the mapped TSSs are ignored, demonstrating that there are on average fewer uORFs in defined 5' UTRs than in untranscribed general intergenic sequences. Although this difference is substantial, it could in part be due to the smaller sample sizes in the true TSS sets and the counting of uORFs in multiple bins for 4149 unmapped sequences. To test this further we considered the uORF density (number of uORFs per 100 nt) in Figure 2B which was calculated for the 4,149 real UTRs compared to 'random' sequences with the same composition. The latter were generated from 100 *n *shuffles (where *n *= length of UTR) for each of the original UTRs to create 100 *n *different sequences and calculating a mean value for the number of uORFs. There is a significant increase in uORF density for shuffled UTRs compared to the real UTRs (t = -34.657, p < 0.001). This shows that there are fewer uORFs than would be expected by chance in sequences of identical composition, lending further weight to this observation. This suggests that the knowledge of the true TSS imposes constraints on the number of uORFs found in upstream sequences, and the properties of uORFs should only be considered when formally located within a mapped 5' UTR sequence – and not just in upstream sequences of a single fixed length.

It should also be noted that there is a correlation between 5' UTR length and the number of uORFs present (Pearson correlation r = 0.746, p < 0.0001). Despite this, a sizeable fraction of 5' UTR sequences have no uORFs at all, including some of those above 400 nt in length when at least three uORFs are expected by chance (see Additional File 2). We examined these genes for common function or role (e.g. common Gene Ontology definitions), but no obvious patterns emerged. Nevertheless, there appears to be a subset of yeast genes which have long 5'UTRs without apparent uORFs suggesting that evolution has selected against uORFs, as one might expect, since they generally act as negative regulators of the standard ORF translation.

### Features of uORFs

#### Coding Frame Bias and uORF length

As a quality control step, we analysed the apparent reading frame of each uORF with respect to that of the true ORF, to test whether there was any frame bias (see Additional File 3). None was found suggesting there is no apparent affect due to misannotation of true start sites or a similar artefact.

**Figure 3:**
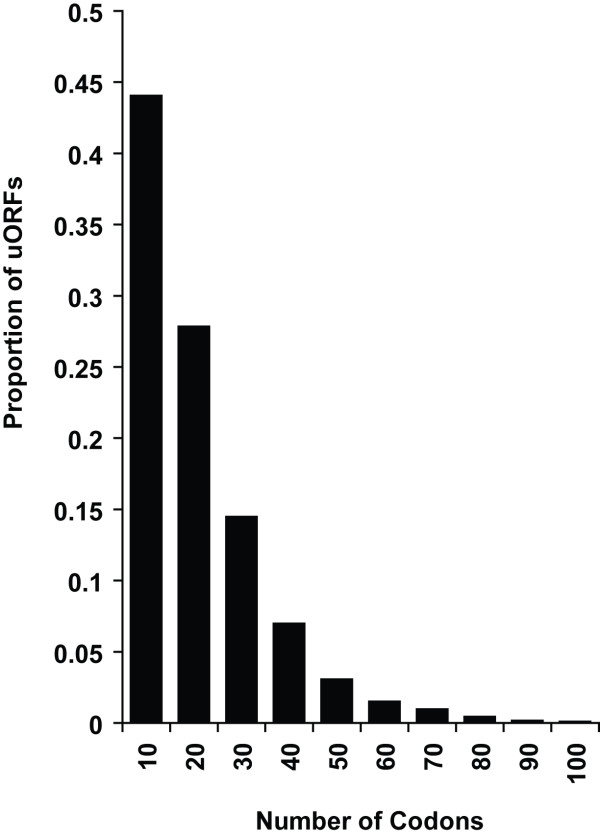
**Size of uORFs in 5' UTRs**. The distribution of uORF lengths in the mapped 5' UTRs is shown; the vast majority are shorter than 20 codons.

As noted in previous analyses, the vast majority of uORFs are short with a mean length of 16 codons (Figure 3), and it has been suggested that short uORFs of 4–6 codons are more likely to have a functional role [28]. Despite this, a significant number are over 50 codons in length and we postulated that some might potentially be misannotations or some other genetic element (disabled ORF or pseudogene). However, none had significant homologues with any protein coding genes when searched against the NR database using BLAST suggesting this is not the case.

It has been reported that uORFs greater than 35 codons in length (105 nt) decrease the reinitiation competence of ribosomes to zero [31]. A total of 151 of the identified uORFs (~10%) exceed 35 codons (105 nt), although this corresponds to the UTRs of only 115 yeast genes (see Additional File 4). Our results suggest that such large uORFs do not necessarily adversely affect general translation of the normal ORF otherwise these genes could not be translated, and hence their uORF contexts may support ribosomal progression and re-initiation.

### uORF visibility and translational efficiency

**Figure 4:**
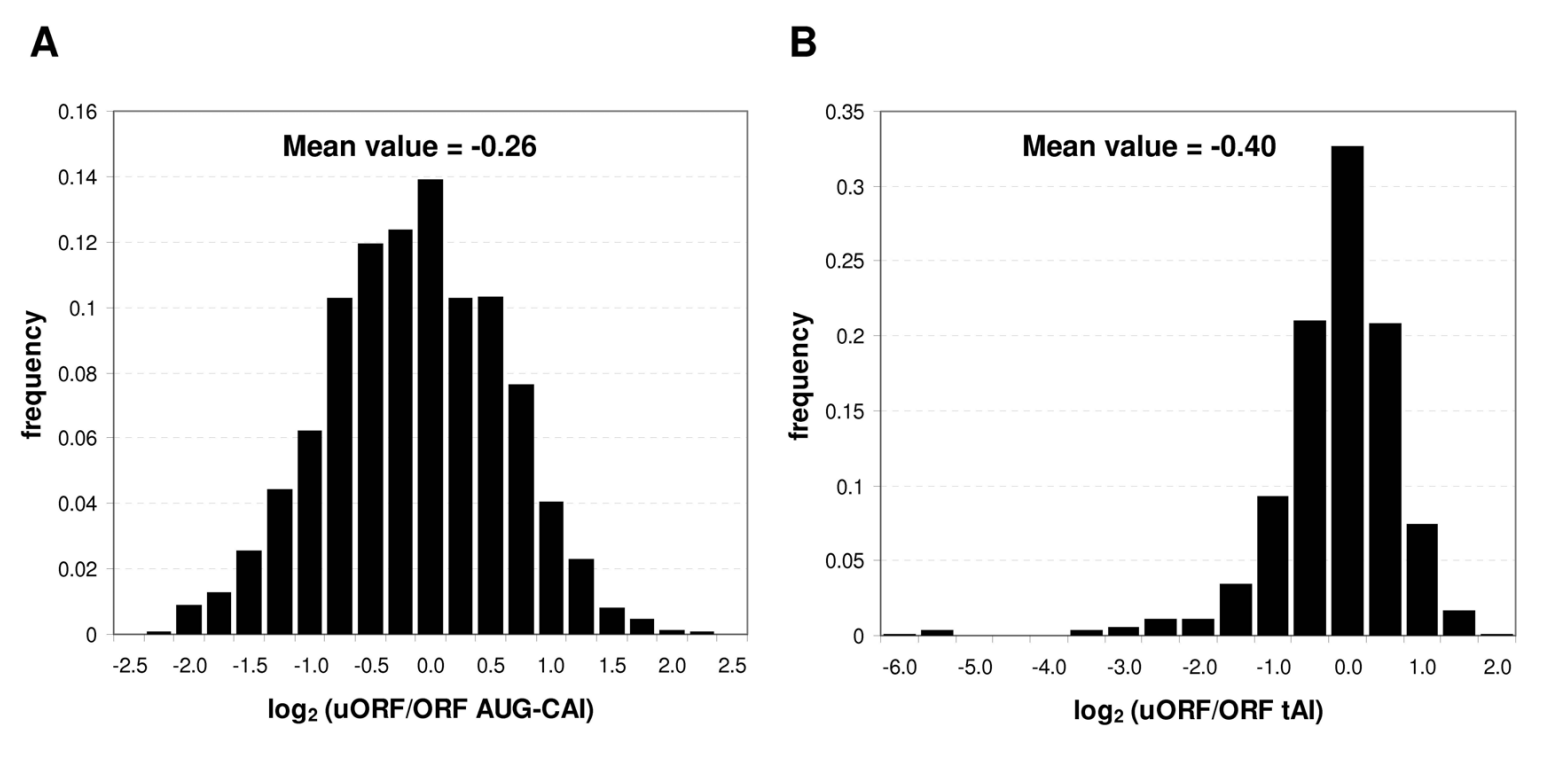
**Relative uORF compositional adaption to the translational machinery**. Panel A shows the frequency histogram of log_2 _ratios of uORF to principal ORF start codon adaption scores (AUG-CAI(r)). If a uORF is better adapted than the principal ORF, it will produce a positive log value. Panel B shows that same comparison considering the translation adpation index (tAI) of the complete reading frame in order to calculate the log_2 _ratio. In both cases, uORFs are considerably less well adapted in general with log_2 _mean values less than zero.

A potential general mechanism for uORFs to affect translational efficiency is as a decoy from the principal ORF, causing premature termination, by triggering mRNA decay via NMD, or delaying the ribosomal complex from attaching and initiating translation at the correct AUG start codon. The potential for a uORF to be recognised by the translational machinery depends upon the sequence composition around the AUG, a concept known as leaky scanning where non-optimal sequences may be bypassed. For this reason the AUG codon adaption index (A_UG_-CAI(r), see Methods) has been proposed [26] to assess the "visibility" of uORFs compared to real ORFs. One might expect uORF starts to be less adapted to the synonymous codon usage of yeast, since they are not principal gene products, and likely to be infrequently translated and unconserved in general [27]. Indeed, this is borne out as the mean A_UG_-CAI(r) score for uORFs is 0.36, lower than the 0.48 for real ORFs. Figure 4A shows a more direct comparison, where we calculate the log_2 _ratio of uORF:ORF A_UG_-CAI(r) for each uORF-ORF pair on the same transcript and plot these values in a frequency histogram. The true ORFs are generally better adapted since most values are less than zero (mean = -0.26). Interestingly, many genes possess at least one uORF that displays a more adapted start codon than the true ORF, leading to a log A_UG_-CAI ratio greater than zero. This is the case for 534 uORF start codons corresponding to 52% of all uORF-containing genes. This suggests that the majority of genes containing at least one uORF possess a uAUG at least as adapted to the translational machinery as the true ORF start. However, we found no systematic bias in this gene subset in terms of GO or other functional category (data not shown).

The A_UG_-CAI(r) only considers the initiation step. Although this is deemed to be rate-limiting to translation [12,32], more general translational efficiency can also be measured by the adaptation of all the uORF's codons to the organism's tRNA pool using the translation adaptation index (tAI) [33]. This metric has shown impressive power in distinguishing phenotypic adaptions across yeast species [34] and is an excellent measure of more general translational competence. We calculated this value for each uORF and each principal ORF and, as expected, principal ORFs are generally more highly adapted than uORFs with mean tAI scores of 0.40 and 0.32 respectively. Again, we calculated log2 ratios of the tAI scores for uORFs and ORFs, shown in Figure 4B. This shows again that principal ORFs appear better adapted with a shift in the distribution towards negative scores. However, a small number of uORFs have very high tAI efficiencies; 64 (4%) uORFS have tAI > 0.6. It should be noted though that these are generally very short uORFs which can skew the tAI calculations compared to large ORFs, and that again, no systematic functional bias in this subset was observed (data not shown). Nevertheless, it has been suggested that shorter uORFs are more functionally significant [28] and one possible route is via translation into bioactive peptides given their high adaptation to the yeast tRNA set.

A final feature to be implicated in translational control is the efficiency of translational release coupled to sequence signals immediately downstream of the stop codon [35,36]. Stop codons normally signal termination during translation and decoupling of the ribosome and translational machinery. However, enhanced stop codon read-through of uORFs could be associated with prolonged ribosomal scanning and have an effect on translational competency. Therefore, we examined uORFs stop signals compared to representative sequences and motifs associated with read-through from previously reported studies [35,36]. These authors define 6-mer motifs immediately downstream of the true ORF stop codons which are either over- or under-represented globally in yeast. Over-represented motifs are expected to be typically efficient stop signals [36], whilst the converse is true of the under-represented motifs. Indeed, under-represented motifs found solely on a statistical basis matched previously known leaky scanning signals and were also confirmed independently [36]. We calculated the frequencies for these two sets of 15 motifs for both uORF and ORF stop contexts, as shown in Table 1. The frequencies observed at ORFs and uORFs are converted to log likelihoods for each motif, highlighting preference of uORF stops with respect to ORF stops. The log likelihoods show that uORFs show a marked propensity to disfavour the strong, common signals located at real ORF stop codons, and they show a preference for 12 out of the 15 "leaky" weak termination signals. This preference is expected given that uORFs with strong signals would promote termination. Similarly, weaker signals close to the true AUG start might preclude sufficiently prompt reinitiation and subsequent translation of the real ORF from taking place. This behaviour is similar to that observed in the well studied *GCN4 *5' UTR, where uORF1 displays the "leaky" UUUxxU motif and positively influences the ability of the ribosome to resume scanning following termination [37]. When low levels of eIF2-GTP bound to Met-tRNA are present, uORF4 is ignored and the main ORF translated. Our data suggests that similar differential scanning effects may be more widespread amongst genes with uORF-containing 5' UTRs, and not just confined to *GCN4*.

**Table 1:** Comparison of stop codon context compositional biases in ORFs and uORFS

	**3' context**	**ORF observed Frequency**	**All uORFs Observed Frequency**	**Log Odd Ratio (uORF/ORF)**
Over-represented 6-mers downstream of stop codons	ATCNNC	0.007478	0.003891	-0.942
	ATCNNT	0.009572	0.003891	-1.299
	AGGNNA	0.009423	0.004669	-1.013
	TCANNT	0.008376	0.007004	-0.258
	GCCNNC	0.003290	0.001556	-1.080
	ATANNA	0.014508	0.011673	-0.314
	GCTNNC	0.002393	0.000778	-1.621
	TAGNNT	0.006132	0.006226	0.022
	ATCNAN	0.016303	0.000778	-4.389
	ACANAN	0.011666	0.010117	-0.206
	AGGNAN	0.008376	0.006226	-0.428
	GCCNAN	0.003889	0.001556	-1.321
	TGANAN	0.010171	0.007004	-0.538
	ATANAN	0.018546	0.010895	-0.767
				
Under-represented 6-mers downstream of stop codons	**CAANNA**	0.003440	0.010117	**1.556**
	**TTTNNT**	0.014059	0.017899	**0.348**
	**AAGNNG**	0.002842	0.004669	**0.716**
	CAANNC	0.001645	0.000778	-1.080
	**CAANNT**	0.003590	0.004669	**0.379**
	**CTCNNT**	0.001346	0.003891	**1.531**
	CATNNT	0.003440	0.000778	-2.144
	**CAANAN**	0.003590	0.010895	**1.601**
	**TTTNTN**	0.013760	0.020233	**0.556**
	**CCGNTN**	0.000150	0.000778	**2.379**
	**CTCNAN**	0.001346	0.001556	**0.209**
	**GATNAN**	0.002991	0.007782	**1.379**
	CAANTN	0.003290	0.003113	-0.080
	**CAANGN**	0.002393	0.003891	**0.701**

The under-represented 6-mers favoured by uORFs are highlighted in bold text.

### Analysis of stress response datasets

After examining the uORF dataset for features and properties that correlate with the TSS mapped UTRs, we cross-referenced this data with gene sets known to exhibit differential expression as a response to different cellular stresses, particularly at the translational level. The aim was to investigate the reasons for translational changes in gene regulation in terms of the TSS-mapped UTR set and uORFs in particular. This data covered four stress response datasets; amino acid starvation, butanol addition, 0.2 M H_2_O_2 _addition and 2 M H_2_O_2 _addition. For each case, changes in both transcriptional and translational expression were characterised using standard array-based technology coupled with polysomal gradient analysis [19,20]. In each of the stress conditions the full complement of yeast genes were initially considered, although not all passed quality control tests from the array analyses [19,20]. Cross-referencing these sets with the TSS-datasets yielded 3,770 genes for amino acid starvation and butanol addition, and 3,860 genes for the H_2_O_2 _additions. The reduction in mapped TSS genes is a result of minor annotation differences between the papers and the Affymetrix GeneChips™.

The TSS filtered stress-response datasets were ranked according to the change in translational state. In the case of amino acid starvation and butanol addition this was characterised as the log ratio of the differential polysomal expression (polysomal P and monosomal M) observed from control (C) to stress (S) conditions (PS/MS:PC/MC). This characterises the translational "shift" in expression in stressed conditions by following the movement of each gene from polysomal to monosomal states. It should be stressed that the nature of these experiments characterise this as a relative change in translational control, since for stresses such as amino acid starvation, there is a general downshift in translation. Given these ratios measure relative changes, the values are not directly comparable between experiments and instead we selected gene lists representing the "extremes" in relative up/down regulation. Where possible, the top and bottom 100 gene log ratios that were above 0.9 and below -0.9, respectively, were used for subsequent analysis; this was possible with all but the butanol addition experiment where only 54 passed this filter (54 up and 54 down). In the subsequent analyses we refer to gene sets as "up" regulated (log ratio > 0.9) and "down" regulated (log ratio < -0.9) where strictly these values characterise differential relative expression states in the stress condition compared to the control state.

**Figure 5:**
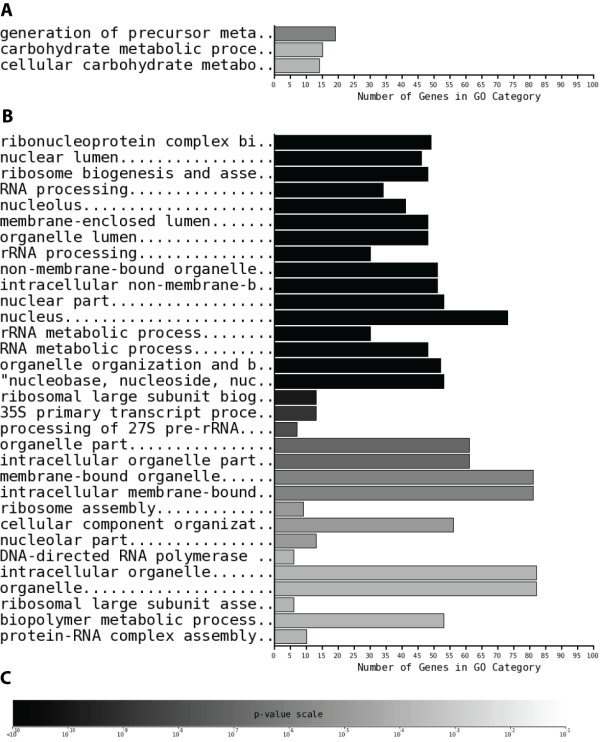
**GoStat over-representation statistics for translationally controlled gene subsets under amino acid starvation**. The numbers of genes in each GO functional category are shown for yeast genes which are differentially translationally regulated under amino acid starvation conditions. Panel A shows significant over-representation for genes which are up-regulated, and panel B for those that are down-regulated. Panel C shows the greyscale key for the associated p-values. Only GO categories with p-values < 0.001 are shown.

We next examined whether any of these differentially regulated gene sets were enriched in specific biological functions. This followed on from our previous analyses where functional biases were observed using hand-curated functional assignments. Here, all of the 8 gene lists were submitted to GoStat [38] in order to find over-represented Gene Ontology (GO) categories. A representative set is shown in Figure 5, showing genes up- and down-regulated translationally under amino acid starvation conditions (see also Additional File 5 for peroxide stresses). In all cases except butanol, significant enrichment in GO categories is observed for the gene sets, with statistical significance assigned using p-values estimated by GoStat [38,39]. These general findings have been discussed in detail in previous papers [19,20], but not in the context of GO categories, and not with formal statistical estimates of significance. Nevertheless, the results are in strong agreement, finding significant enrichment in expected GO functional categories. For example, genes that are down-regulated under amino acid starvation stress are over-represented in GO categories relating to regulation of ribosomal and RNA processing. Genes that are post-transcriptionally up-regulated in 0.2 mM H_2_O_2 _are over-represented in stress adaptation and protection GO categories, and down regulated genes are enriched in ion transport and mitochondrial-related GO annotations. For 2 mM H_2_O_2 _addition, GO categories for RNA processing and ribosomal biogenesis are over-represented in the up-regulated genes set and oxidoreductase activity is over-represented in the down regulation set. The only stress without significant GO over-representation was butanol addition. This was likely due to the smaller number of genes that satisfied the log ratio cut-off values in this stress condition; butanol stress exerts a general translational effect on only a specific, small number of genes [19]. Related observations have been made by other studies [40-43]. For example, the shift from glucose to glycerol and rapamycin addition both cause similar translational down-regulation of protein synthesis genes in general and ribosomal proteins in particular in common with our results.

Our data suggest that a significant proportion of the yeast proteome appears to be, in part, regulated at the translational level, particularly under stress conditions. Given this observation, we wished to examine the transcript sequences of the genes involved to try and explain the differential regulation in terms of known control mechanisms. Namely, these were uORF-based attenuation of translation, known functional 5' UTR motifs, and the propensity of 5'UTRs to form secondary structures, all of which have been proposed as general translational control mechanisms [5,6,44]. Although post-transcriptional control mechanisms other than these exist, the availability of transcriptional data to define comprehensive 5' UTR data sets, as well as 3' UTR data [23], make them attractive to study. Additionally, 5' UTRs are expected to play a major role since they are recognised and scanned by the translational machinery.

### 5'UTR length and uORF analysis of stress response genes

**Figure 6:**
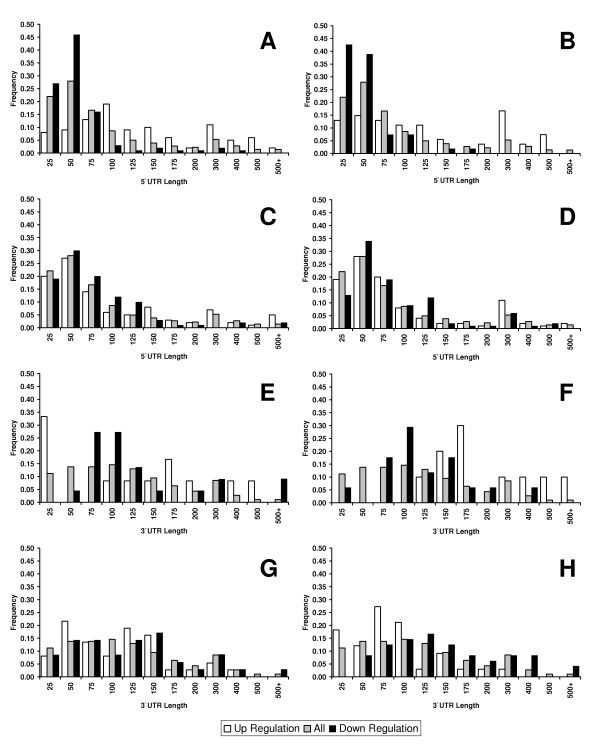
**Histograms of UTR lengths for translationally controlled gene sets**. The 5' UTR lengths are shown for gene sets that are up- or down- regulated under varying stress conditions. A. Amino acid starvation, B. Butanol addition, C. 0.2 mM H_2_O_2_, D, 2 mM H_2_O_2_., along with 3' UTR length distributions for gene sets under E. Amino acid starvation, F. Butanol addition, G. 0.2 mM H_2_O_2_, and H, 2 mM H_2_O_2_

As indicated, the differential translational regulation of yeast genes is likely to involve UTR sequence, and consequently we analysed the 5' and 3' UTR lengths of genes which were post-transcriptionally up and down regulated under various stresses, shown in Figures 6A–H. As can be seen, there is a trend towards longer 5'UTRs in translationally up regulated genes and shorter 5'UTRs in translationally down regulated genes. These trends are reproduced using the 5'TSSs defined by the tiling arrays (Additional File 6). Similarly, trends are observed in the 3'UTR data. In general, they are longer than 5'UTRs, as previously reported [23]. The 3'UTR results show some general agreement with the 5'UTR data for the amino acid and butanol data (up-regulated genes have longer UTRs than down-regulated), but the opposite trends are observed for both H_2_O_2 _addition experiments. These data suggest that they also play an important, but different, role to 5'UTRs in regulating translation [23]. As shown in Table 2 the majority of these UTR length differences are statistically significant with a bias for longer 5'UTRs in the up-regulated genes and shorter 5'UTRs for down regulated genes, when compared to all genes. The exception was 2 mM H_2_O_2 _addition with no significant length differences found for 5'UTR lengths, although there is a significant difference for 3'UTR lengths which are shorter in up-regulated genes (Table 2). Our previous results suggested high H_2_O_2 _concentrations promote polyribosomal association for some genes [20] and an absence of mRNAs with associated localisation signals in their UTRs could be linked to this.

**Table 2:** T-test results for UTR length differences in all four stress conditions

**5' UTRs^a^**						
	*Up v Down*		*Up v All*		*Down v All*	
	longer/shorter	P-value	longer/shorter	P-value	longer/shorter	P-value
Amino Acid	**Longer**	**<10^-3^**	**Longer**	**<10^-3^**	**Shorter**	**<10^-3^**
Butanol	**Longer**	**<10^-5^**	**Longer**	**0.007**	**Shorter**	**<10^-3^**
0.2 mM Peroxide	**Longer**	**0.001**	**Longer**	**0.010**	**Shorter**	**<10^-3^**
2 mM Peroxide	Longer	0.437	Longer	0.685	Shorter	0.413

**3' UTRs^b^**						
	*Up v Down*		*Up v All*		*Down v All*	

	longer/shorter	P-value	longer/shorter	P-value	longer/shorter	P-value
Amino Acid	Longer	0.430	Longer	0.248	Longer	0.091
Butanol	**Longer**	**0.003**	**Longer**	**<10^-4^**	Shorter	0.078
0.2 mM Peroxide	Shorter	0.140	Shorter	0.160	Longer	0.355
2 mM Peroxide	**Shorter**	**0.005**	**Shorter**	**0.013**	**Longer**	**<10^-3^**

^a ^calculations were performed between the stress response gene subsets cross-referenced with all 4,129 5' TSS mapped genes.

^b ^calculations were performed between the stress response gene subsets cross-referenced with the 2,044 3' UTR defined in [23].

**Figure 7:**
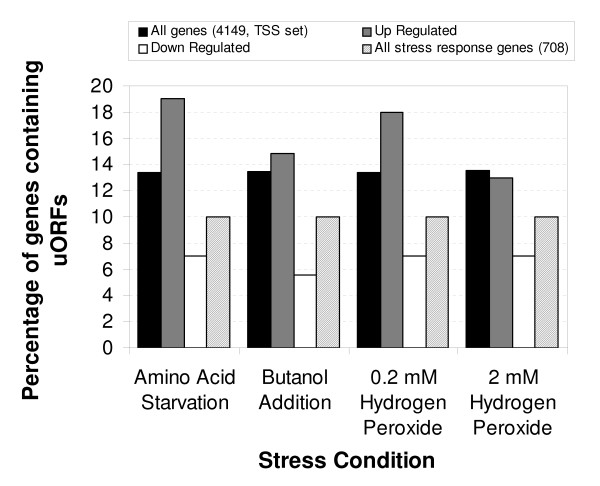
**Proportion of uORF-containing genes in translationally controlled gene sets**. The fraction of different gene sets whose UTRs contain uORFs are shown for different stress conditions labelled on the x-axis. This is compared to all 4149 TSS-mapped genes and all 708 genes showing differential regulation in any of the 4 stress conditions. A general trend of uORF enrichment is observed in the up-regulated gene sets compared to down-regulated under all stresses. Up and down-regulated genes were excluded from the "All Genes" set.

Other studies have also considered transcript length in the context of translational efficiency and gene function [8,45]. Arava and colleagues used ribosomal density mapping to discover an anti-correlation between transcript length and ORF length, although this was in unstressed cells [8]. However, this did not include the precise knowledge of the 5'UTR length. Similarly, Hurowitz and Brown [45] noted that shorter than expected transcripts were observed in several GO classes including ribosomal proteins. Both these results are consistent with trends observed here, namely that transcript length (in our case 5'UTR length) is linked to translational control, and that different functional classes of genes are enriched for shorter/longer UTR lengths. The ribosome density mapping data suggests that in unstressed cells, longer transcripts have fewer ribosomes and therefore might be less efficiently translated. Our data suggests the converse under stress where longer UTRs have a role to play in escaping the general down-regulation of translation. However, quite how this is achieved is uncertain. Indeed, at first glance, the results for 5'UTRs are perhaps somewhat at odds with expectation. If uORFs are generally acting as translational "decoy" elements which reduce translational efficiency, then genes with longer UTRs/more uORFs ought to be down-regulated. Here, however, we show the opposite effect; genes with longer UTRs escape from the general down-regulation under stress and are up-regulated with respect to genes in general. This suggests that this "escape" mechanism relies on UTR content and that genes with short or negligible UTRs do not contain sufficient information or content to avoid down regulation. We also considered the percentage of genes containing uORFs in the differentially translated gene sets shown in Figure 7. The proportion of genes that contain uORFs in the 5'UTR is much greater for post-transcriptionally up-regulated genes than down-regulated genes under all stresses and than all genes under all but the high H_2_O_2 _stress. There are two caveats to this though; uORFs are relatively rare and, it has not been established whether the increase in uORF frequency is solely a consequence of longer UTRs. Indeed, it is entirely possibly that 3'UTRs and other 5' features/motifs are playing a role, particularly since uORFs are relatively rare. It is also worth noting that the level of uORFs in the set of 708 stress response genes generated by combining all the up/down-regulated gene lists for all four stresses is generally lower than average at ~10% (Figure 7).

These data provide further strong evidence that UTRs, and uORFs in particular, are involved in mediating translational control over yeast genes, particularly under stress conditions. Also, and perhaps surprisingly, they allow many genes to overcome the general down regulation observed. This mechanism has been observed before under stress, for *GCN4 *[37] and our data suggest that it may be more widespread in translational responses to stress.

### Conservation of uORFs

To investigate the uORF presence in UTRs further, we considered whether they were conserved across closely related species, namely *Saccharomyces cerevisiae *and 6 *sensu stricto *yeast species. Previous studies have already examined conservation of uORFs in yeast concluding that in general only a limited number are conserved, and a smaller subset still are deemed of functional importance [27,28]. However, these studies pre-dated the recent TSS datasets and did not cross-reference the data with differential translational control in stress conditions. Nevertheless, they both highlight the difficulties concerned in defining conservation for these small genetic elements. Should they be absolutely conserved in UTRs to exert their effects (in position and/or composition), or is merely the presence of one or more uORFs anywhere within the 5' UTR sufficient? There is no simplistic single measure which accounts for all considerations. Here we have attempted to consider both, first calculating a direct measure of conservation at each single position in the *S. cerevisiae *UTR, using the phastCons program [46]. This approach formally considers the relative evolutionary distance between the member genomes and yields a single value for each aligned position in a multiple sequence alignment. The phastCons score ranges from 0 to 1 (absolutely conserved). Secondly, we calculated a Z-score for each uORF, as the number of standard deviations from the mean score of similar sized windows in each UTR.

We considered a dataset of 61 "ultra-conserved" uORFs in more detail which had phastCons score > 0.99 and were completely conserved in at least 1 other species (see Additional File 7 for a complete list). To examine whether cross-species conservation was in part an artefact of the high phastCons score, we estimated the average level of conservation observed for randomly selected short UTR sequences with high phastCons scores, comparing them with the aligned sequence in the other species for conservation. This was done by selecting subsequences of the pattern C1-X_n_-C2, where codons C1 and C2 are separated by *n *codons, where *n *> 0. The average phastCons score for these subsequences was calculated, as well as the number of species in which the pattern was "conserved". Since these test sequences are not uORFs, "conservation" is defined in a way analogous to a small ORF. The first codon C1 must be completely conserved (analogous to a start codon), the C2 codon must code for the same amino acid (analogous to conserving a stop signal) and C1 and C2 must be in-frame in the aligned species. The average number of "conserved" species was then calculated for phastCons scores in the range 0.99 to 1.

**Figure 8:**
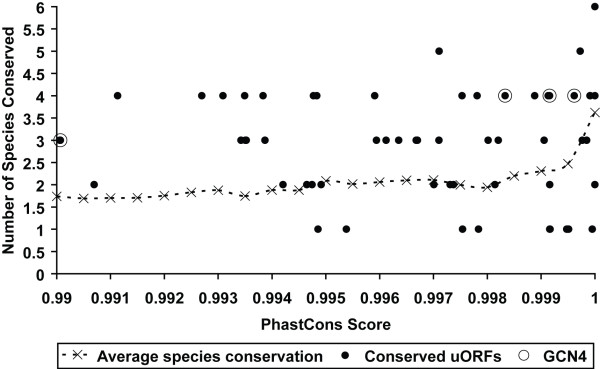
**Conservation statistics for highly conserved uORFs**. For individual uORFs with mean phastCons scores greater than 0.99 (highly conserved regions) across 6 Sensu Stricto yeast species, the phastCons score is plotted against corresponding number of species (out of 6) in which the uORF is conserved. This is shown with respect to an "average" species conservation metric comparing sequences with similar properties to an open reading frame (see Methods). These 61 uORFs show considerable conservation, both in terms of local sequence context (phastCons scores) and the number of species which also share a uORF, and includes previously characterised uORFs from the well known GCN4 gene, shown as circled dots.

The results for all these various measures of conservation are shown in Figure 8, which shows the average number of species conserved and phastCons scores for highly conserved uORFs with phastCons scores 0.99 to 1. Data are only shown for uORFs with a Z-score > 2 above that expected by chance for the given UTR (where Z-score is the number of standard deviations above the mean phastCons score for equivalently sized subsequences in 5' UTR sequence; See Methods). These 61 uORFs, taken from 43 genes, therefore represent a subset that is highly conserved both in terms of absolute and relative conservation of their UTRs, and in terms of specific conservation of the uORF in closely related species. Indeed, the majority of the uORFs are more conserved than random (the dotted line) in Figure 8. This dataset of uORFs extends the set of 15 discovered previously [27] which are highly conserved and suggests that there is a larger number than previously known which demonstrate significant functional significance. Our criteria for conservation are strict, and there are a large number of uORFs with high conservation (196 have phastCons scores > 0.9 with Z-scores > 2). Notably, 7 out of 17 of the uORFs from the TSS mapped Zhang & Dietrich set [27] appear in this 196 uORF subset which corresponds to 126 genes. The remaining 10 were filtered out at an early stage since their gene did not have a mapped TSS. This demonstrates that our alternative strategy to measure conservation shows general consistency. It is also interesting to note that all 4 *GCN4 *uORFs satisfy all our criteria and are conserved in more species than expected; all 4 uORFs are present and circled in Figure 8, mirroring previous studies using other approaches [27,28]. If we assume the 196 uORF/126 gene set are functionally important due to their conservation, cross-referencing with those previously annotated [27,28] yields 365 genes – a significant proportion of the genome.

We also examined the correspondence between these 61 conserved uORFs and up/down regulation in our stress response gene sets. Unfortunately, only 4 of these genes appeared in the stress response data sets and it is therefore difficult to draw any general conclusions. However, one notable gene is the mitochondrial alcohol dehydrogenase *ADH3 *(YMR083W) which has a highly conserved uORF at position -281 with respect to the start codon, and is down regulated in low peroxide concentrations.

## Conclusion

In this study we have examined the role of upstream sequence elements in yeast UTRs, considering whether they play a concerted role in regulation of gene expression under stress conditions. Specifically, we considered available data sets of full length mapped yeast transcripts to define a superset of mapped transcriptional start sites (TSSs) representing over 70% of yeast protein coding genes. This is the most comprehensive survey of yeast uORFs using known 5' UTR sequence, and the first time this data has been considered in light of known translationally regulated genes under stress conditions [17,18]. The analysis shows that yeast uORFs are statistically under-represented in 5' UTRs and UTRs have generally evolved to select against uORFs, suggesting that those that are tolerated may play some specific role or function in translation. This is in agreement with previous studies, either on specific genes [13,14] or more generally [6,11,27-29] which have suggested functional roles for uORFs. In addition to their relative scarcity, they are also less "efficient" in terms of their start codon local sequence (A_UG _CAI index), their overall codon bias (tAI), and importantly, their preferred stop codon contexts. The decreased translational adaption of uORFs has also been noted for those associated with NMD [26] and is consistent with their scarcity in UTRs and generic functional role. They would be expected to reduced translation efficiency of the principal ORF, blocking the ribosome from progressing to the true ORF or even promoting termination and detachment [26]. Interestingly however, they tend to avoid strong stop signals which would promote termination, instead allowing re-initiation of the ribosomal machinery to continue scanning. One possibility is that uORFs might permit ribosomes to stall and "wait", a general mechanism which has been suggested to facilitate a fast response once stresses have been removed and normal translation can then continue [20].

Regarding differential regulation at the translational level, a significant trend is observed across most of the stresses examined here. Genes which are observed to undergo relative translational up-regulation under stress have longer UTRs. This observation seems self-evident when considered at face value – namely, that any gene which can be regulated at the translational level must have a mechanism to support this, and this should be *via *some motif or element contained within either the 5' or 3' UTR. However, here we demonstrate this for a variety of stresses for the first time, and importantly, demonstrate that this is a statistically significant trend. It offers a simple approach to select genes which are more likely to be translationally regulated on the basis of the UTR size and contents. Interestingly, the recent tiling array study of the yeast genome also defined 5' UTRs for a subset of the gene set [23] and these authors also noted trends with 5' UTR length. They noted that anecdotally, genes with shorter 5' UTR lengths were generally "housekeeping" genes involved in processes such as rRNA metabolism, RNA processing and ribosomal biogenesis. Our polysomal array data supports this, finding that these Gene Ontology categories are translationally down-regulated under stress, and do not have longer 5' UTRs that allows them to escape this. Equally, we also demonstrate genes with longer 5' UTRs are translationally up-regulated and includes those involved in processes such as transport and localisation as reported by David and colleagues [23]. This provides further evidence for a relationship between mRNA transcript length and gene function as proposed by Hurowitz and Brown [45].

Pinpointing the precise nature of the elements conferring translational regulatory properties is rather more challenging. Our data suggests that uORFs play a significant role mediating gene expression during stress responses, as they are over-represented in translationally up-regulated genes, particularly under 0.2 mM peroxide stress. However, this trend is not so striking as the UTR length correlation, and must in part be a result of this; longer UTRs are more likely to have a uORF.

It should also be noted that the over-representation in up-regulated genes is difficult to reconcile with the "standard" uORF mechanism where they are generally expected to down-regulate gene expression at the translational level. This suggests that they are either acting in a novel way, that the complex "GCN4"-type mechanism is more widespread, or that other UTR elements than uORFs are responsible. Regardless, given their relative scarcity it is clear that there is still much to learn about UTR and uORF function.

Other authors have focused on conservation as a strong predictor of functional significance [27,28,46,47]. Although early studies have suggested that uORFs are generally not conserved, this is a far from straightforward calculation to make. A single uORF may not necessarily be conserved in terms of exact sequence, length or relative position with respect to either the transcriptional or translational start, yet might still fill its functional role. In this study we add the additional constraint of known transcriptional start site and consider two complementary conservation metrics, the phastCons score [46] and a Z-score local conservation statistic looking at the local UTR background. We have also examined whether a uORF is directly conserved in close fungal relatives. Although uORFs are generally not conserved, many are more conserved than by chance within their respective UTR sequences, confirming and extending previous studies [27,28]. Using strict criteria, we find 61 uORFs (from 43 genes) with high conservation across related fungal species (See Additional File 7) extending these previously reported data sets to 365 genes.

It has been reported that secondary structure in 5' UTRs mediates translational control of gene expression on a genomic scale in yeast [5,44]. We re-examined this result using the true TSS-mapping for the 4149 5'UTR sequences and the program Randfold [48]. In broad agreement with Ringnér and Krogh [44] the vast majority of 5'UTRs appear not to be strongly folded; only 20 5'UTRs were found to have low MFE values with an associated p-value < 0.005 (see Additional File 8). However, we do not see the same general trend between calculated 5' UTR folding energies and translation rates. Clearly, the use of the true TSS has a marked effect on the 5' UTR folding energy and raises the possibility that this trend might also be a result of the true size of the UTRs. Put simply, shorter 5' UTRs facilitate faster translation. Nevertheless, the secondary structural states might be stronger not weaker in the true 5' UTRs and this also seems to be a significant regulatory mechanism for a small number of genes.

In summary, the results presented here demonstrate convincing evidence that 5'UTR sequence has a major role to play in the regulation of gene expression, particularly under stress conditions and at the translational level. This effect appears to be widespread, affecting large numbers of different yeast genes under different conditions. Yeast has evolved a variety of mechanisms to effect these changes, including upstream open reading frames which are over-represented in translationally up-regulated genes.

## Methods

### Sequences

All *S. cerevisiae *open reading frame (ORF) chromosomal co-ordinate information was obtained from the SGD website via:  and all *S. cerevisiae *sequences were obtained from 

### Transcription start and end sites

Transcription start site (TSS) information was obtained from published studies, for 5' UTRs from Zhang and Dietrich [21], and from Miura and colleagues [22] via supplementary data . The two gene sets were merged by ORF name selecting a single TSS for each ORF. For each ORF with multiple mapped TSSs, the occurrence of each TSS was counted. If the modal TSS for each ORF had a count of greater than two it was taken as the representative site for that gene, otherwise the longest 5'UTR was selected, the most distal from the ATG start codon. The 5'UTRs sequences were then taken from the ATG start codon up to the chosen TSS. A complete list of uORFs and 5' UTRs is given in Additional File 9. In addition, the 3' UTR ends were taken for a high quality 2,044 gene subset of the yeast genome determined via tiling arrays [23].

### Stress response datasets

Microarray data pertaining to the stress response gene sets were obtained from ArrayExpress  using accession numbers E-MEXP-323 (amino acid starvation), E-MEXP-324 (butanol addition) and E-MEXP-526 (0.2 mM and 2 mM H_2_O_2 _additions). Translational up- and down-regulation under stress for each gene was calculated as previously described [19,20] using log ratios of shifts between polysomal (P, highly translated) and monosomal (M, weakly translated) components to monitor changes in translational control between stress (S) and control (C) experiments. In the H_2_O_2 _stress experiments PS/MS:PC/MC was not used as the sole measure of translational state, due to oxidative stress inhibiting ribosomal transit [20]. In addition, the sum of the monosomal and polysomal fractions for stress conditions was compared to control conditions (PS+MS:PC+MC). For the peroxide experiments, up regulated genes were defined as genes that satisfied both criteria, with PS/MS:PC/MC and PS+MS:PC+MC greater than 1. Similarly, down regulated genes had PS/MS:PC/MC and PS+MS:PC+MC less than 1. This serves to highlight genes that when "up regulated" were able to overcome the translational initiation block and the inhibition of ribosomal transit.

### A_UG_-CAI(r) and TAI indices

A previously published method was implemented [26] taking the look-up values from a position specific weight matrix calculated for positions (-6,-5,-4,-3,-2,-1,4,5,6) of the AUG initiation context of 63 highly expressed genes. The A_UG_-CAI is then calculated for each candidate AUG context by calculating the geometric mean of these look-up values (multiplying the 9 weights together and taking the 9^th ^root). This mean value is then normalised, dividing by the maximum theoretical value, to give an A_UG_-CAI(r) value between 0 and 1.

The translation adaption (tAI) index implements a similar method, originally developed by dos Reis and colleagues [33,34] (available from ). This estimates codon weights from gene copy numbers of the tRNA isoacceptors for a given codon, essentially reflecting the relative ease with which a gene can be translated based on the availability of the necessary tRNAs for that codon. A look-up table of codon adaptation weights were calculated for *S. cerevisiae *genes were and subsequently used to calculate a geometric mean for a given open reading frame as described in ref [33].

### Gene Ontology over-representation

Over-representation statistics of Gene Ontology categories within gene-sets were performed using GOStat [38] at  using yeast SGD gene names of the respective gene sets as input.

### Defining upstream open reading frames

Upstream open reading frames (uORFs) were identified in 5'UTRs and up to 100 nucleotides into the real open reading frame (ORF). We defined uORFs from the relative nucleotide positions and coding frame of all start and stop codons with respect to the ATG start of the true ORF. All in-frame uORFs were collected from this set, taking the longer of any subsets that contained that same stop codon position. All identified uORFs were discarded if they were either less than three codons in length (including the stop codon) or within 20 nt of the 5'end, as the latter are not considered to have an effect on translation [6].

### Randfold analysis

Predicted secondary structure with 5'UTR sequences was calculated using the Randfold method [48]. All 5'UTR sequences were submitted to Randfold using the dinucleotide method and 100 L randomizations, where L = length of 5'UTR.

### PhastCons analysis

Conservation of individual base positions in each UTR was calculated using related fungal genomic sequences aligned to *S. cerevisiae*, using the PhastCons [46] algorithm. Alignment blocks and PhastCons scores for *S. cerevisiae *genomic alignments against *S. paradoxus, S. Mikitae, S. kudriavzevii, S. bayanus, S. castelli and S. kluyveri *where obtained using the Table Browser at UCSC [49], and individual phastCons scores extracted for each position. PhastCons scores were obtained from the UCSC table browser for only 3,877 of the 4,149 5'UTRs due to incomplete alignments covering the UTR region in *S. cerevisiae*. The average phastCons scores were calculated for all 3,877 uORFs across the all positions from start to stop, inclusive. An average "background" phastCons score was also calculated for each uORF from the 5'UTR sequence containing it, and the uORF scores converted to Z-scores. This provides an additional measure of whether a uORF is conserved over and above the level of conservation observed generally in each UTR, In addition, multiZ genomic alignment fragments were also obtained from the UCSC browser for 90 highly conserved uORFs with a mean phastCons scores >0.99. The aligned species sequences were analysed for in-frame start and stop codons that corresponded to those of the *S. cerevisiae *uORFs.

## Authors' contributions

SJH, MA, GP, CG conceived and designed the study, with help from all authors. CL carried out all the computational analyses on UTRs, gene function and uORFs, under the guidance of SJH with the following exceptions; RP carried out an initial analysis on upstreamAUGs/uORFs in fixed upstream segments, JNS carried out the processing of microarray data and generation of candidate gene lists. JS carried out all the experimental microarray work. CL and SJH wrote initial drafts of the manuscript, all authors offered comments and have read and approved the final manuscript, edited by SJH.

## Supplementary Material

Additional file 1 **Comparison of UTR lengths in two datasets.** Comparison of 5' and 3' UTR lengths from the dataset generated for this study with that of David et al's tiling arrat study [23]. Click here for file

Additional file 2 **Observed and expected frequencies of uORFs in mapped 5' UTRs.** This file contains a figure showing the distribution of observed and expected frequencies of uORFs in 5'UTRs Click here for file

Additional file 3 **Observed reading frame distribution of uORFs in mapped 5' UTR.** Figure showing the relative frequency of uORF open reading frames with respect to the main ORF reading frame. For those uORFs wholly contained within the 5' UTR there is clearly no bias towards any frame suggesting that there are no likely missannotations of the true ORF start or independent ORFs. Click here for file

Additional file 4 **Summary statistics for long uORFs greater than 35 amino acids in length.** This Excel format file file contains statistics for individual uORFs (one per line) whose length exceeds 35 amino acids. For each uORF, the ORF, ORF length, 5' UTR length, frame and start position are indicated, as well as the total number of uORFs contained in the given 5' UTR. Click here for file

Additional file 5 **GoStat over-representation statistics for translationally controlled gene subsets under H2O2 stress.** This Figure shows similar over-representation statistics as shown in Figure 5 but for both the butanol stress conditions. Click here for file

Additional file 6 **UTR length distributions for different gene sets under stress conditions using tiling array definitions of TSS.** Length distributions equivalent to those shown in Figure 6 but using the subset of genes defined by tiling array studies which overlap the differential regulation data sets generated for yeast stress responses used in this study. Click here for file

Additional file 7 **Full Statistics for conserved 61 uORF sub set.** This spreadsheet provides the full data for conserved uORFs, listing the parent gene, position of uORF in the UTR, PhastCons score, conservation Z-score, and number of aligned species in which a uORF is directly conserved. Click here for file

Additional file 8 **Analyses of 5' UTR secondary structures using RanFold.** Tabulated statistics of Randfold calculations are shown for the 5 most likely secondary structure containing UTRs along with potential secondary structure diagrams. Click here for file

Additional file 9 **Complete statistics for all uORFs and UTRs used in the study.** This details all the uORFs from all UTRs used in this study, providing locations, parent genes, and sequence information, as well as the details on all the 5' UTRs used, and their start sites with respect to each named yeast gene. Click here for file
