# Supplementary material for: Upstream sequence elements direct post-transcriptional regulation of gene expression under stress conditions in yeast
Source: BMC Genomics. 2009 Jan 7;10:7. doi: 10.1186/1471-2164-10-7 (PMC2649001; doi:10.1186/1471-2164-10-7)
Supplement: Additional file 1 — Comparison of UTR lengths in two datasets. Comparison of 5' and 3' UTR lengths from the dataset generated for this study with that of David et al's tiling arrat study [23]. [file 1471-2164-10-7-S1.doc]

**Additional File 1.**

**A**

**B**

**Figure S1. Comparison of UTR lengths in two datasets.** The 5’ UTR lengths from the tiling array study of David et al [23] and used in this study (from the vector-capping and SAGE data) are plotted for genes present in both datasets. In A, the data are shown for 5’ UTRs only, with a clear correlation of 0.56 observed. Notably, many genes show a perfect match and are present on the leading diagonal whilst others differ substantially. This is in part due to the nature of the data where a single TSS has been selected to represent one gene. The tiling array data is deemed to be an “average” from a high quality array signal of the probes at the boundary, whilst the data generated here will represent a single value selected from a small number of candidates. Clearly many yeast genes are transcribed from different starts and this has led to disparity for a significant number. In B, here it can be clearly seen that there is no apparent relationship between 5’ and 3’ UTR length which agrees with the general result in [23] although here we take the 5’ and 3’ data from different sources.
