# Supplementary material for: Upstream sequence elements direct post-transcriptional regulation of gene expression under stress conditions in yeast
Source: BMC Genomics. 2009 Jan 7;10:7. doi: 10.1186/1471-2164-10-7 (PMC2649001; doi:10.1186/1471-2164-10-7)
Supplement: Additional file 2 — Observed and expected frequencies of uORFs in mapped 5' UTRs. This file contains a figure showing the distribution of observed and expected frequencies of uORFs in 5'UTRs [file 1471-2164-10-7-S2.doc]

**Additional File 2.**


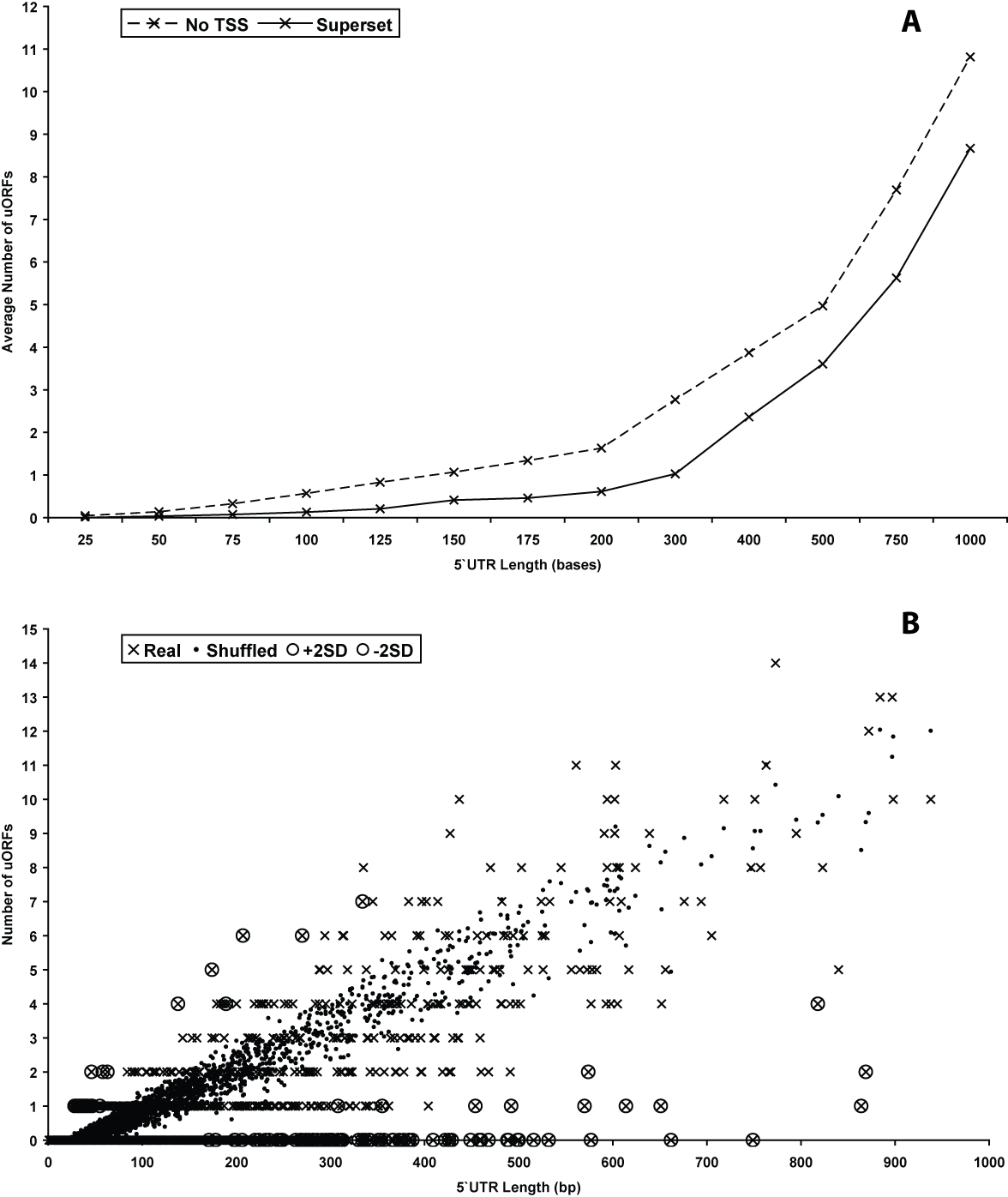


**Figure S2a. Observed and expected frequencies of uORFs in mapped 5’ UTRs.**

The number of uORFs in mapped 5’ UTRs is plotted against 5’ UTR length for both real and shuffled UTRs for the 4129 TSS mapped yeast genes. The true number of uORFs is plotted as a cross in Figure S1. This is placed into the context of the number of uORFs expected by chance for each gene, by shuffling the sequence of each UTR and recalculating the number of uORFs. A single point is shown in Figure S2a for each UTR, calculated as the average number of uORFs from 100*n* shuffles (where *n* = length of UTR). Many UTRs contain more or fewer uORFs than would be expected by chance with those outside 2 standard deviations from the expected mean highlighted with a circle (31 genes above and 78 genes below the expected mean). However, this is not corrected for multiple testing and also assumes the expected number of uORFs is normally distributed. In fact, as shown in Figure 2B, there are generally fewer uORFs in UTRs than would be expected by chance. Indeed, as shown in Figure S2a a large fraction of genes have none at all, including some of those above 400 nt in length when at least three uORFs are expected by chance. Conversely, there are no genes with > 2 standard deviations from the mean value for UTRs greater than 400 nt in length. This further supports the case that uORFs are rare and avoided, since they would generally interfere with the standard fidelity of translation. It also shows that the distribution of uORFs is skewed towards lower values and is hence not normal (since 5% of the data would otherwise be expected to be above and below 2 standard deviations from the mean value).

**Figure S2b. Observed and expected uORFs densities in mapped 5’ UTRs and matched length upstream sequences.**

This shows the data in Figure 2A from a slightly different perspective, using uORFdensity (x100) as the comparative statistic. To try and mitigate any potential bias in the counting of uORFs in the mapped/unmapped upstream sequences, UTRs are binned into length groups and the average uORF density calculated. Interestingly, this statistic increases with UTR length which is presumably due to the greater chance of observed a short open reading frame in a sequence of increasing length. This is due to the requirement to observe both a start and stop codon in frame and in close proximity. However, the equivalent matched length sequences (plotted as open squares above) show an increased density throughout, which begins to tail off around 300 nucleotides. At all lengths up to this threshold, true TSS mapped UTRs have a consistently lower uORF density which appears to increase roughly linearly with UTR length. At longer UTR lengths, this increase tails off in both the real and expected data. In the case of the TSS mapped UTRs there are considerably fewer longer UTRs (above 500 nt) and therefore less significance in the points.
