# Supplementary material for: Upstream sequence elements direct post-transcriptional regulation of gene expression under stress conditions in yeast
Source: BMC Genomics. 2009 Jan 7;10:7. doi: 10.1186/1471-2164-10-7 (PMC2649001; doi:10.1186/1471-2164-10-7)
Supplement: Additional file 3 — Observed reading frame distribution of uORFs in mapped 5' UTR. Figure showing the relative frequency of uORF open reading frames with respect to the main ORF reading frame. For those uORFs wholly contained within the 5' UTR there is clearly no bias towards any frame suggesting that there are no likely missannotations of the true ORF start or independent ORFs. [file 1471-2164-10-7-S3.doc]

**Additional File 3**


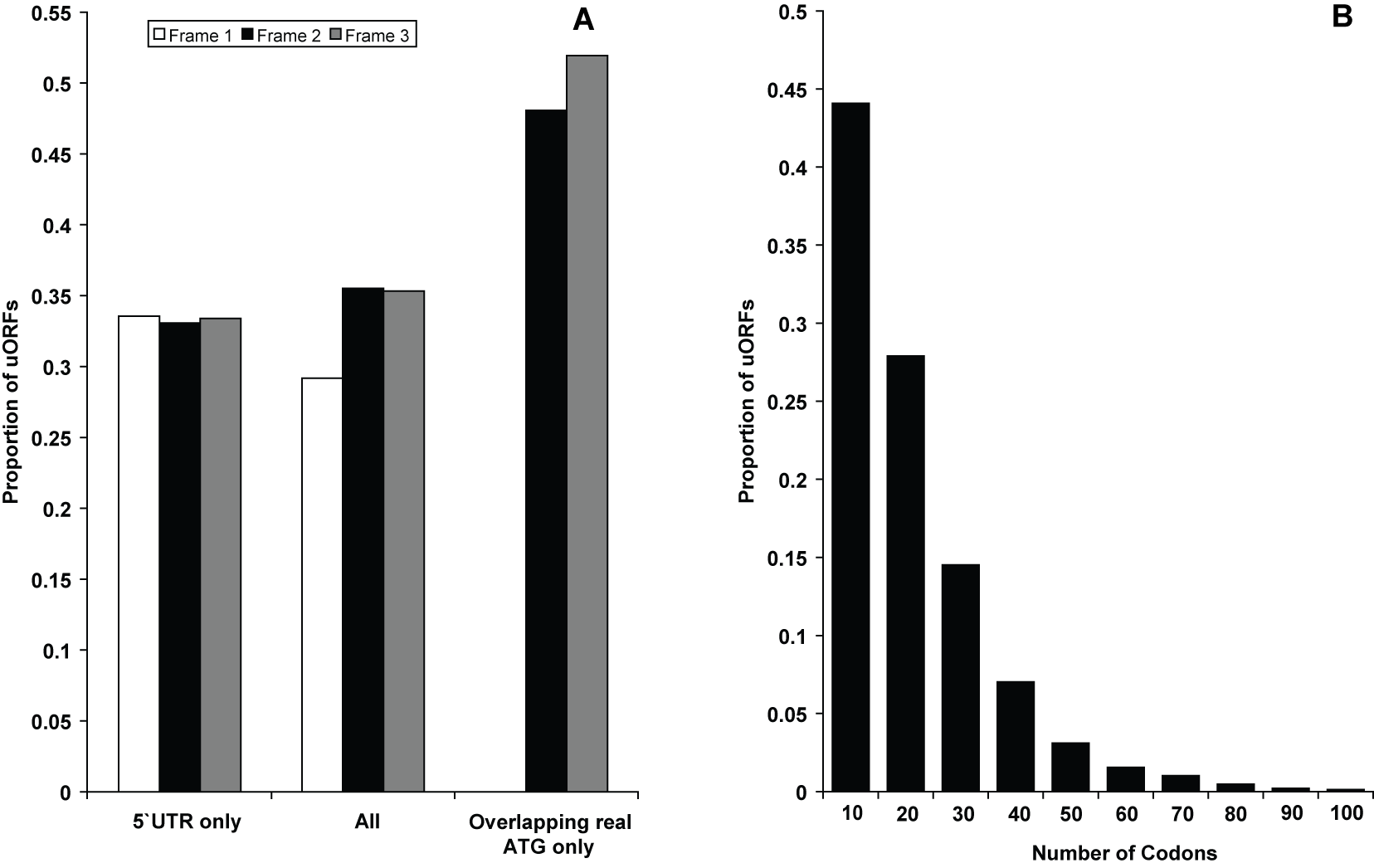


**Figure S3. Observed reading frame distribution of uORFs in mapped 5’ UTRs.**

The frequency distribution of uORFs in all three coding frames was calculated to assess if there was a bias for a particular frame, shown above in Figure S3. When taking into account uORFs that do not extend into the first 100 nt of the real ORF there is an even distribution across all three reading frames. In the subset of all uORFs that do extend across the start codon there are no uORFs in frame 1 - uORFs which were in frame 1 would constitute alternative start sites if they over-lapped the true ORF AUG. When combined, there is therefore an apparent small bias against frame 1 in the “all” set, although this is due to the aforementioned bias in the AUG-overlap set. There is therefore no real apparent bias for any particular reading frame with respect to the true ORF.
