# Supplementary material for: Upstream sequence elements direct post-transcriptional regulation of gene expression under stress conditions in yeast
Source: BMC Genomics. 2009 Jan 7;10:7. doi: 10.1186/1471-2164-10-7 (PMC2649001; doi:10.1186/1471-2164-10-7)
Supplement: Additional file 5 — GoStat over-representation statistics for translationally controlled gene subsets under H2O2 stress. This Figure shows similar over-representation statistics as shown in Figure 5 but for both the butanol stress conditions. [file 1471-2164-10-7-S5.doc]

**Additional File 5**


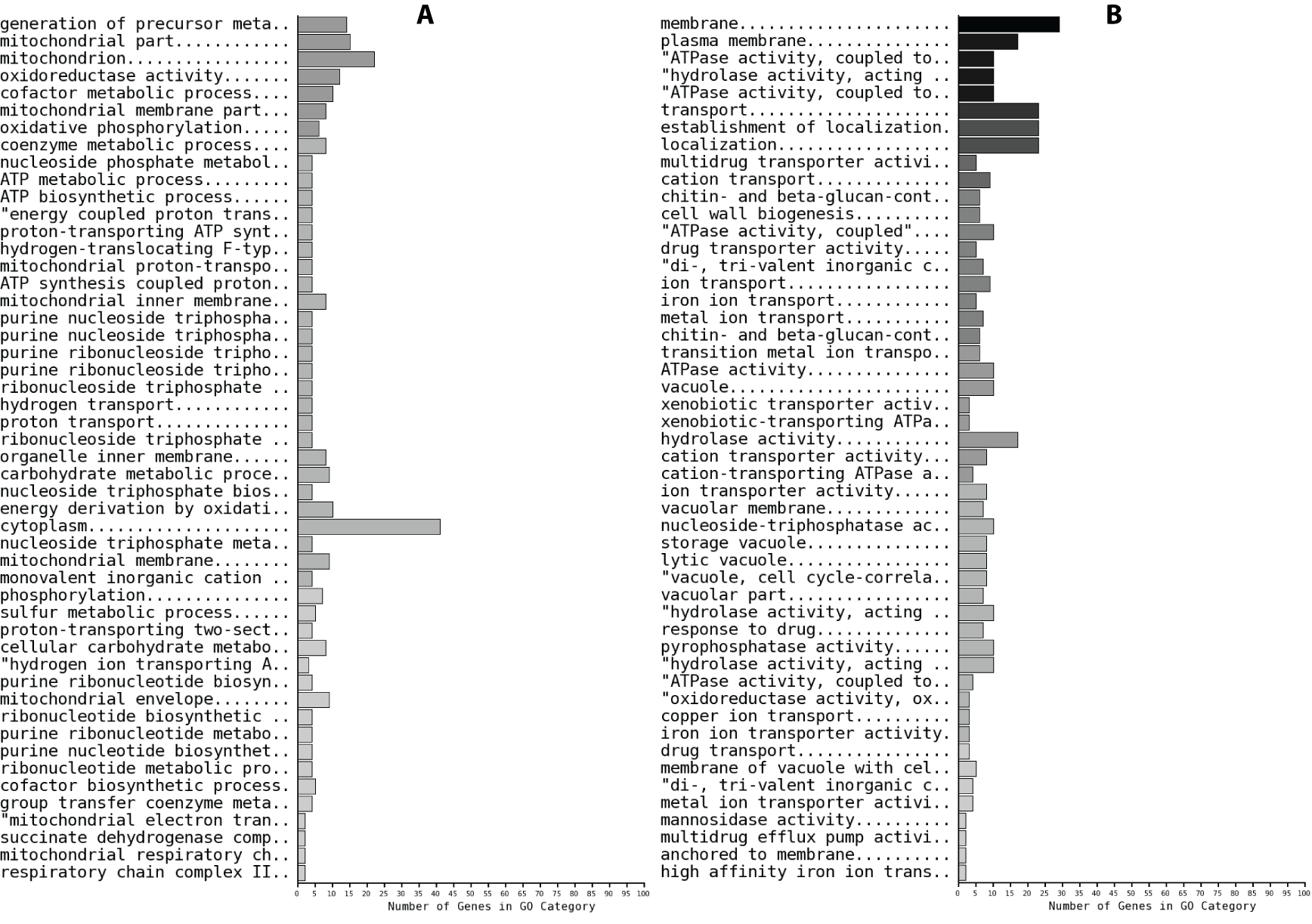


**D**

**C**


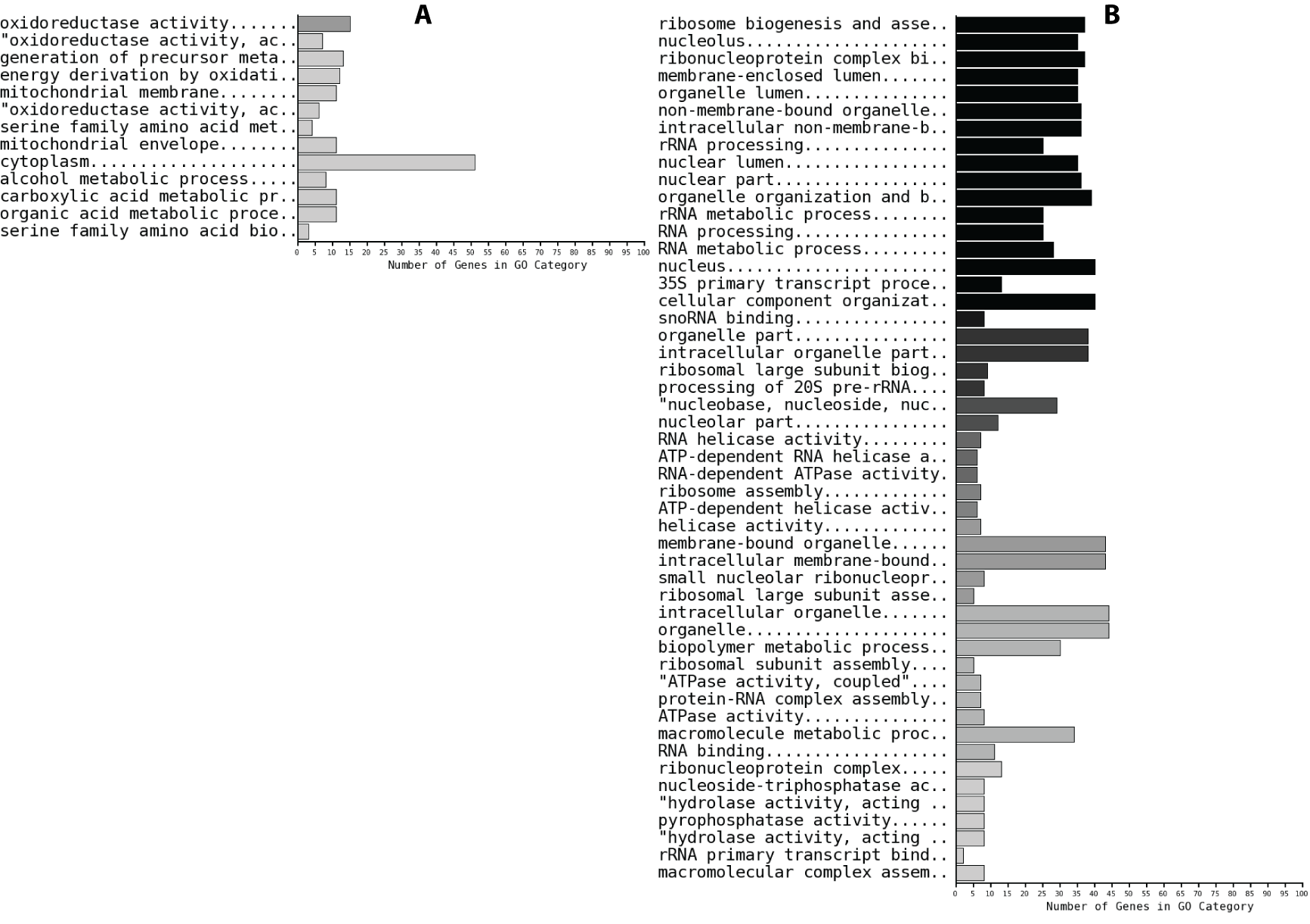


**E**

**
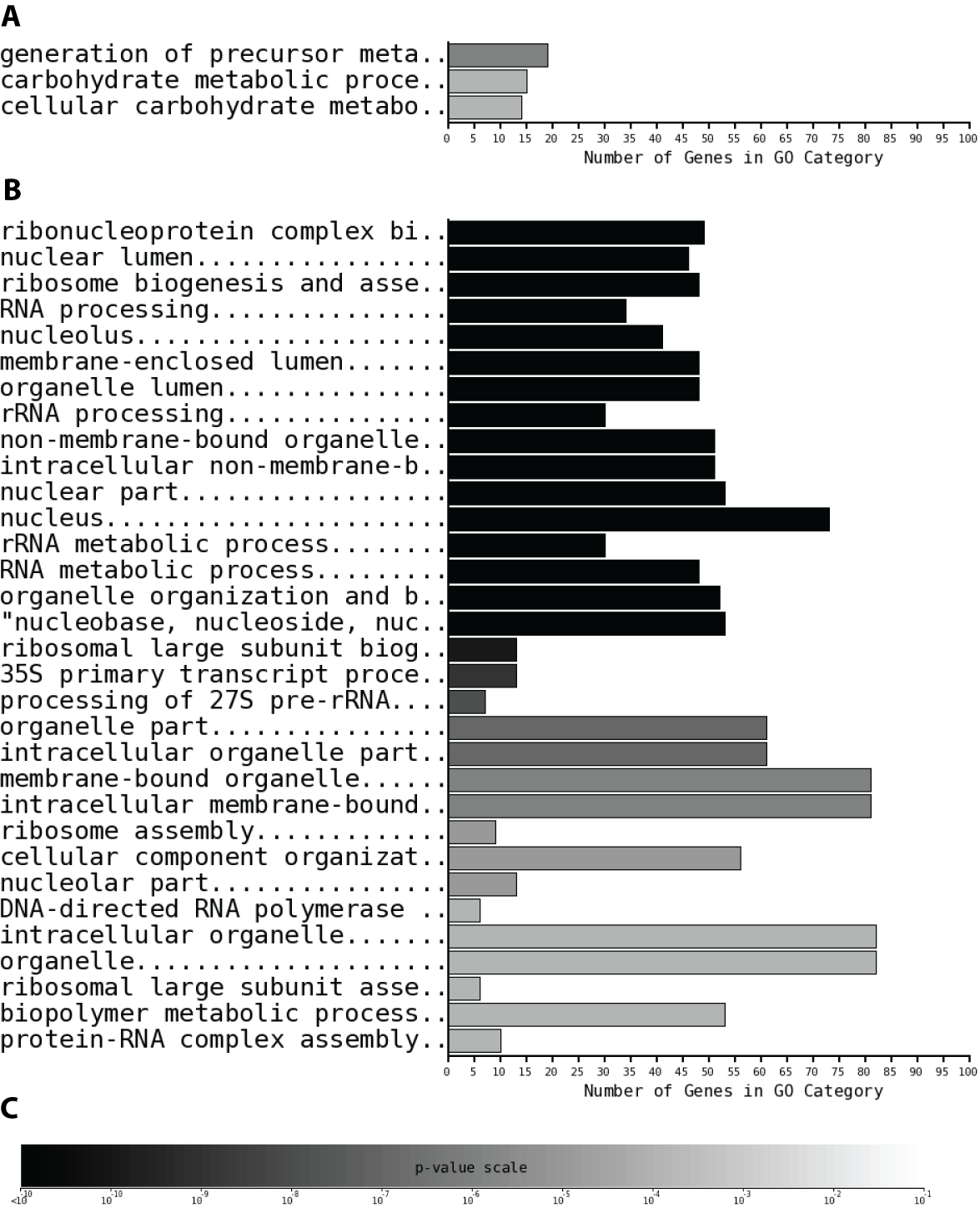
**

**Figure S4. GoStat over-representation statistics for translationally controlled gene subsets under H_2_O_2_ stress.** The numbers of genes in each GO functional category are shown for yeast genes which are differentially translationally regulated under 0.2mM peroxide stress (A,B) and 2.0mM peroxide stress (C,D) conditions. Panels A and C shows significant over-representation for genes which are up-regulated, and panels B and D for those that are down-regulated. Panel E shows the greyscale key for the associated p-values. Only GO categories with p-values < 0.001 are shown.
