# Supplementary material for: Upstream sequence elements direct post-transcriptional regulation of gene expression under stress conditions in yeast
Source: BMC Genomics. 2009 Jan 7;10:7. doi: 10.1186/1471-2164-10-7 (PMC2649001; doi:10.1186/1471-2164-10-7)
Supplement: Additional file 6 — UTR length distributions for different gene sets under stress conditions using tiling array definitions of TSS. Length distributions equivalent to those shown in Figure 6 but using the subset of genes defined by tiling array studies which overlap the differential regulation data sets generated for yeast stress responses used in this study. [file 1471-2164-10-7-S6.doc]

**Additional File 6.**

**A**

**C**

**D**

**B**

**Figure S5. UTR length distributions for different gene sets under stress conditions using tiling array definitions of TSS.** These figures correspond directly to those shown in Figure 6, although the tiling array data of David et al [23] were used to define 5’ starts. We used the data corresponding to those transcripts for which confident 5’ and 3’ ends could be identified. When cross-referenced with the stress response gene sets showing differential regulation this yielded smaller sets than for the larger 4149 gene set of 5’ UTR starts used for Figure 6, but the trends are very similar – the only exception is 2 mM H_2_O_2_ but the different in mean length is not statistically significant in either case. Due to the smaller nature of the tiling array set, the statistics were not so significant in general, although the trends are the same. A, amino acid starvation, B, butanol, C, 0.2mM H_2_O_2_, D, 2.0 mM H_2_O_2_.The data is shown in Table S5 below.

**Table S1. T-test results for 5’ UTR length differences in all four stress conditions**

| **5’ UTRs^a^** | | | | | | |
| --- | --- | --- | --- | --- | --- | --- |
|  | *Up v Down* | | *Up v All* | | *Down v All* | |
|  | longer/  shorter | P-value | longer/  shorter | P-value | longer/  shorter | P-value |
| Amino Acid | **Longer** | **<10^-4^** | **Longer** | **<10^-3^** | **Shorter** | **0.005** |
| Butanol | **Longer** | **<10^-4^** | **Longer** | **<10^-5^** | Shorter | 0.14 |
| 0.2mM Peroxide | **Longer** | **0.02** | Longer | 0.09 | Shorter | 0.11 |
| 2mM Peroxide | Longer | 0.27 | Longer | 0.439 | Longer | 0.256 |

^a^ calculations were performed between the stress response gene subsets cross-referenced with the 2,044 5’ UTRs defined in David *et al* [23].
