# Supplementary material for: Upstream sequence elements direct post-transcriptional regulation of gene expression under stress conditions in yeast
Source: BMC Genomics. 2009 Jan 7;10:7. doi: 10.1186/1471-2164-10-7 (PMC2649001; doi:10.1186/1471-2164-10-7)
Supplement: Additional file 8 — Analyses of 5' UTR secondary structures using RanFold. Tabulated statistics of Randfold calculations are shown for the 5 most likely secondary structure containing UTRs along with potential secondary structure diagrams. [file 1471-2164-10-7-S8.doc]

**Additional File 8**

**Analyses of 5’ UTR secondary structures using RanFold**

Predicted secondary structure within 5’ UTR sequences was calculated using the Randfold method (see main paper for references). All 5`UTR sequences were submitted to Randfold using the dinucleotide method and 100 L randomizations, where L = length of 5`UTR. In general, very few of the 5’ UTRs showed a strong likelihood to form secondary structure. Indeed, only 20 had significantly low predicted MFEs with associated p-values < 0.0005. The top-5 most significant results for predicted MFE across the whole UTR are shown below in Table S3. Note that compared to fixed length segments leading up to the ATG start, the known 5’ UTRs (used in this study) have very different predicted values to those obtained when the true 5’ start site is known. This has implications for any general conclusions drawn from studies which do not know the true 5’ start, and base their calculations on fixed lengths upstream from the ATG start codon.

**Table S2: The MFE scores for the five 5`UTR secondary structures with the lowest p-value. Previously calculated MFE scores for the same UTRs when TSS was unknown are all shown.**

| **Gene** | **Name** | **Ringnér 50nt** | **Ringnér 100nt** | **Ringnér 200nt** | **Randfold MFE** | **pvalue** | **5`UTR Length** | **comments** |
| --- | --- | --- | --- | --- | --- | --- | --- | --- |
| YDL122W | UBP1 | -0.01 | -11.22 | -13.92 | -50.02 | 4×10^-5^ | 241 | Ubiquitin-specific protease that removes ubiquitin from ubiquitinated proteins; cleaves at the C terminus of ubiquitin fusions irrespective of their size; capable of cleaving polyubiquitin chains |
| YGL215W | CLG1 | 0.0 | -8.9 | -10.31 | -102.3 | 2×10^-4^ | 468 | Cyclin-like protein that interacts with Pho85p; has sequence similarity to G1 cyclins PCL1 and PCL2 |
| YGL008C | PMA1 | -2.1 | -2.9 | -5.69 | -42.9 | 2×10^-4^ | 236 | Plasma membrane H+-ATPase, pumps protons out of the cell; major regulator of cytoplasmic pH and plasma membrane potential; part of the P2 subgroup of cation-transporting ATPases |
| YPL210C | SRP72 | -3.4 | -17.7 | -19.15 | -38.9 | 2×10^-4^ | 131 | Core component of the signal recognition particle (SRP) ribonucleoprotein (RNP) complex that functions in targeting nascent secretory proteins to the endoplasmic reticulum (ER) membrane |
| YMR265C | - | -7.9 | -13.4 | -16.49 | -132.94 | 6×10^-4^ | 440 | Uncharacterised |
